# In Situ Determination of Nitrate in Water Using Fourier Transform Mid-Infrared Attenuated Total Reflectance Spectroscopy Coupled with Deconvolution Algorithm

**DOI:** 10.3390/molecules25245838

**Published:** 2020-12-10

**Authors:** Fangqun Gan, Ke Wu, Fei Ma, Changwen Du

**Affiliations:** 1 The State Key Laboratory of Soil and Sustainable Agriculture, Institute of Soil Science Chinese Academy of Sciences, Nanjing 210008, China; qunfanggan@163.com (F.G.); fma@issas.ac.cn (F.M.); 2 College of Environment and Ecology, Jiangsu Open University, Nanjing 210017, China; kwu@issas.ac.cn; 3 College of Advanced Agricultural Sciences, University of Chinese Academy of Sciences, Beijing 100049, China

**Keywords:** nitrate, water bodies, Fourier transform attenuated total reflection, deconvolution, curve-fitting, partial least squares

## Abstract

Fourier transform infrared attenuated total reflectance (FTIR-ATR) spectroscopy has been used to determine the nitrate content in aqueous solutions. However, the conventional water deduction algorithm indicated considerable limits in the analysis of samples with low nitrate concentration. In this study, FTIR-ATR spectra of nitrate solution samples with high and low concentrations were obtained, and the spectra were then pre-processed with deconvolution curve-fitting (without water deduction) combined with partial least squares regression (PLSR) to predict the nitrate content. The results show that the typical absorption of nitrate (1200−1500 cm^−1^) did not clearly align with the conventional algorithm of water deduction, while this absorption was obviously observed through the deconvolution algorithm. The first principal component of the spectra, which explained more than 95% variance, was linearly related to the nitrate content; the correlation coefficient (*R*^2^) of the PLSR model for the high-concentration group was 0.9578, and the ratio of the standard deviation of the prediction set to that of the calibration set (*RPD*) was 4.22, indicating excellent prediction performance. For the low-concentration group model, *R*^2^ and *RPD* were 0.9865 and 3.15, respectively, which also demonstrated significantly improved prediction capability. Therefore, FTIR-ATR spectroscopy combined with deconvolution curve-fitting can be conducted to determine the nitrate content in aqueous solutions, thus facilitating rapid determination of nitrate in water bodies with varied concentrations.

## 1. Introduction

The main forms of nitrogen in aquatic ecosystems are total nitrogen, ammonium nitrogen (NH_4_^+^–N), and nitrate nitrogen (NO_3_^−^–N and NO_2_^−^–N) [1]. In recent years, excess nitrogen in water bodies, especially NO_3_^−^–N, has led to major ecological problems [2]. In addition, NO_3_^−^–N in drinking water can be converted to NO_2_^−^–N by the commensal bacteria in the mouth and digestive tract, which is harmful to the health of adults and children. Their long-term consumption can cause, for example, cancer of the digestive and excretory systems [3,4]. Therefore, there is a need to develop techniques that rapidly detect NO_3_^−^–N in water bodies to prevent water eutrophication and promote human health management.

Conventional methods for measuring NO_3_^−^–N in water include reduction distillation, colorimetry, and the use of ion-specific electrodes [5,6], which are time-consuming and tend to produce secondary pollution. As a fast and nondestructive analysis method, infrared spectroscopy has many advantages, such as a simple analytical process, low cost, high efficiency, and no chemical reagent consumption [7,8,9]. It has recently been used to rapidly determine nitrate nitrogen levels in water. Previous studies have indicated that mid-infrared attenuated total reflection (FTIR-ATR) spectroscopy can be conducted for the rapid quantitative analysis of nitrate in solutions. The results show that the intensity of the characteristic absorption peak of N-O vibration in nitrate (1200–1500 cm^−1^) was proportional to the NO_3_^−^–N concentration. They used this relationship to establish a partial least squares (PLS) model that predicted the nitrate nitrogen content [10]. Shaviv et al. used FTIR-ATR to determine NO_3_^−^–N in deionized water and in soil solutions [11]. Although these studies used FTIR-ATR to detect nitrate nitrogen in water, it was problematic to determine NO_3_^−^–N with low concentrations (such as lower than 20 mg L^−1^) in aqueous solutions due to the significant interference from strong water absorption.

In previous studies, for soil solution and vegetable samples with high concentrations of NO_3_^−^–N, direct water deduction was generally used to remove the interference [12,13,14]. However, for the spectral analysis of low-concentration nitrate samples, water deduction causes large errors. Therefore, the spectral data must be pre-processed effectively to obtain useful information. Deconvolution is a mathematical procedure and a signal processing method typically conducted in many fields such as pattern recognition, seismology, system identification, electromagnetic scattering, and tomography [15]. The application of deconvolution in spectral processing has also proven to be effective. Deconvolution techniques can be used to enhance the resolution beyond the instrumental limit and significantly improve the signal-to-noise ratio [16,17]. In addition, to obtain useful, accurate and reliable information, spectral deconvolution could be associated with the Gaussian fit of the absorption spectra to adjust the Gaussian mathematical curves and obtain the corresponding characteristic absorption from overlapped peaks in a complex spectrum [18,19].

Thus, the objective of this study was to use FTIR-ATR to rapidly determine both high and low concentrations of nitrate in aqueous solutions through the spectra pretreatment of deconvolution curve-fitting, combined with principal component analysis (PCA) and partial least squares regression (PLSR), which could provide a new alternative option for the rapid determination of varied nitrate concentrations in water.

## 2. Materials and Methods

### 2.1. Materials

The test reagents were KNO_3_ (analytical reagent grade, AR, purchased from Nanjing Ronghua Apparatus Co., Ltd., Nanjing, China) and deionized water. High and low nitrate concentrations (NO_3_^−^–N) were prepared separately, in which the high-concentration group included concentrations of 0, 5, 10, 20, 30, 40, 50, 60, 70, 80, 90, and 100 mg L^−1^ and the low-concentration group included 0, 1, 2, 4, 6, 8, 10, 12, 14, 16, 18, and 20 mg L^−1^.

### 2.2. Spectra Recording

An FTIR-ATR instrument (Nicolet 6700) was used (Thermo Fisher Scientific, Waltham, MA, USA), with a DTG detector, and the attenuated total reflection accessory was a 45 °C ZnSe ATR (Bruker, Karlsruhe, Germany). When recording the FTIR-ATR spectra, the nitrate solutions were directly added to the ATR crystal tank and the nitrate solution of each concentration was measured four times. The spectral scan range was set to 500–4000 cm^−1^ and 32 repeated scans were continuously recorded, with a resolution of 4 cm^−1^ and a mirror velocity of 0.4747 cm s^−1^.

### 2.3. Pretreatment of Spectral Data

#### 2.3.1. Water Deduction

The FTIR-ATR spectra were pre-processed with a Savitzky–Golay filter to reduce noise and improve the signal-to-noise ratio [20,21]. MATLAB 2016a (The MathWorks, Natick, MA, USA) was used to deduct the absorption peaks of water with the reference band (wavenumber range of 1500–2200 cm^−1^); then, PCA and PLS analysis were subsequently conducted.

#### 2.3.2. Deconvolution Curve-Fitting (without Water Deduction)

For all solutions, smoothing, baseline correction, and deconvolution curve-fitting (Gaussian) of the spectra within the range 1200–1500 cm^−1^ were performed through the Peakfit 4.12 software (SeaSolve Software Inc., San Jose, CA, USA). The objective of deconvolution was to separate each peak from the comprehensive information in the spectrum [22,23]. The details of the deconvolution process are demonstrated in the supplementary materials (Figures S1–S3). Briefly, the principles are as follows:

*Y*(*x*) = Σ*F_i_*(*x*) (1)

where *Y* is the spectrum; *x* is the wavenumber; *i* (1, 2, 3, … *n*) is the number of isolated peaks; *F* is the expansion function or the kernel function of deconvolution. The Gaussian function is used as the kernel function:

(2) $$y=\frac{a_{0}}{\pi \sqrt{\pi a_{2}}}exp\left[-\frac{1}{2}{\left(\frac{x-a_{1}}{a_{2}}\right)}^{2}\right]$$

where *a_0_*, *a*_1_, and *a*_2_ represent the peak amplitude, position, and width, respectively, and *x* and *y* are the wavenumber and absorption intensity, respectively.

### 2.4. Model Evaluation

The following equations were used to calculate *RMSE*, *RPD*, and *R*^2^ in order to evaluate the performance of the models in the validation set as following:

(3) $$RMSE=\sqrt{\frac{1}{n}\overset{n}{\underset{i=1}{\sum }}{\left(y_{i}-{\hat{y}}_{i}\right)}^{2}}$$

(4) $$RPD=\frac{SD}{RMSE}$$

(5) $$R^{2}=1-\frac{\sum_{i=1}^{n}{\left(y_{i}-{\hat{y}}_{i}\right)}^{2}}{\sum_{i=1}^{n}{\left(y_{i}-\bar{y}\right)}^{2}}$$

where $y_{i}$ and ${\hat{y}}_{i}$ are the measured and predicted nitrate levels of *i*^th^ samples, respectively, $\bar{y}$ is the mean of the measured nitrate, and *n* is the number of samples. High values of *R*^2^ and *RPD* along with a low *RMSE* value indicated a robust and accurate model. *RPD_V_* values of <1.4 were poor; ≥1.4 and <1.8 were fair and allowed the model prediction to be used for assessment and correlation; ≥1.8 and <2.0 were good, in which case quantitative predictions were possible; ≥2.0 and <2.5 were very good for quantitative analysis; and ≥2.5 were excellent [24,25].

## 3. Results and Discussion

### 3.1. FTIR-ATR Spectra of Nitrate

**Figure 1:**
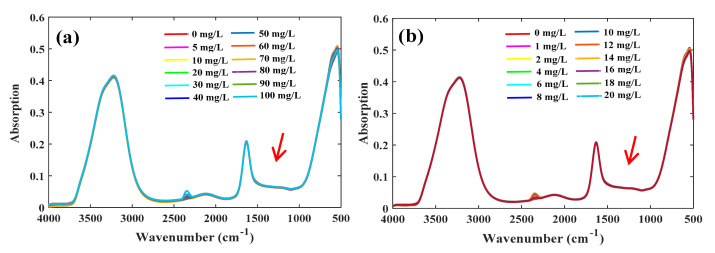
Fourier transforms mid-infrared attenuated total reflectance (FTIR-ATR) spectra of nitrate solutions. (**a**) High-concentration group; (**b**) low-concentration group.

The FTIR-ATR spectra of the high- and low-concentration groups of nitrates showed the same spectral appearance (Figure 1). Two strong absorption peaks appeared in the range of 3000–3800 and 1500–1800 cm^−1^, which are characteristic absorptions of water, indicating that absorptions by water greatly interfered with the absorptions of nitrate in the spectra. The characteristic absorptions of nitrate appeared in the range 1200–1500 cm^−1^, but it was difficult to observe directly because its intensity was much weaker than that of water.

**Figure 2:**
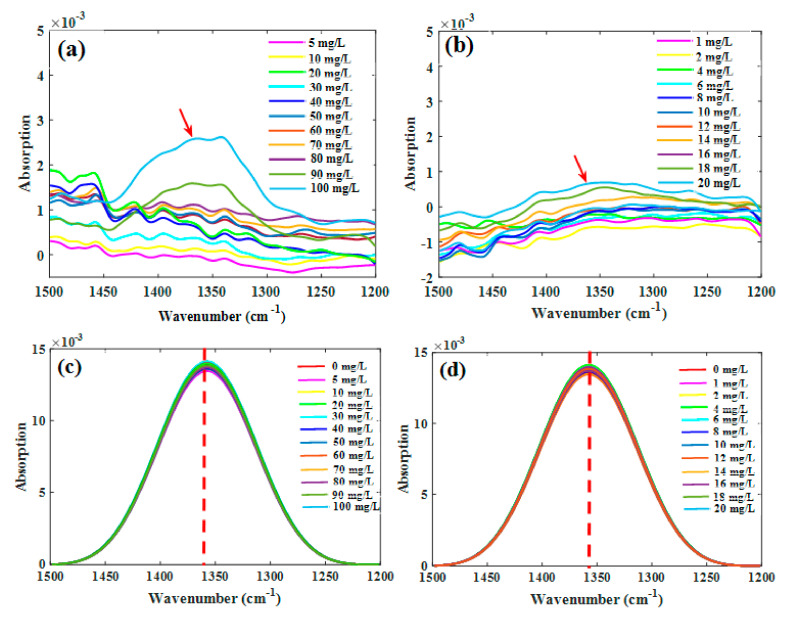
Characteristic absorption bands of nitrate solutions through water deduction ((**a**) high-concentration group; (**b**) low-concentration group; *n* = 44) and deconvolution from raw spectra ((**c**) high-concentration group; (**d**) low-concentration group; *n* = 48).

The spectra of the nitrate solutions of the two concentration groups, after deducting the signal arising from water, are shown in Figure 2a,b. For both the groups, the characteristic peak intensities at different nitrate concentrations did not follow a consistent trend, which mainly resulted from the interference of water absorption. The spectra ranging from 1200 to 1500 cm^−1^ (Figure 2c,d) were then deconvoluted, and the absorption intensity of NO_3_^−^ was visually proportional to the NO_3_^−^–N concentration; therefore, the characteristic peaks within this range could be used for the quantitative analysis of NO_3_^−^–N in solutions. Comparing the nitrate spectra obtained with the water deduction and deconvolution (without water deduction), it showed that deducting water could not effectively reduce the signal interference, while deconvolution could significantly extract the characteristic peaks of nitrate.

### 3.2. Principal Component Analysis

**Figure 3:**
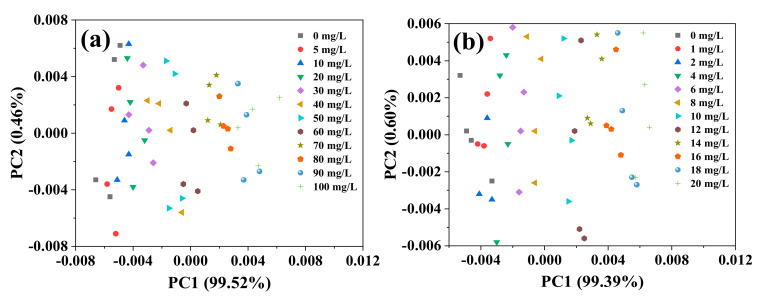
Principal component distribution of FTIR-ATR spectra of the nitrate solution after deconvolution ((**a**) high-concentration group; (**b**) low-concentration group).

PCA was conducted on the spectra within the range 1200–1500 cm^−1^. For high- and low-concentration groups, the first two principal components of both the concentration groups accounted for more than 80% of the spectral information within the range 1200–1500 cm^−1^. Thus, PC1 and PC2 can be used to represent variations in the spectra. However, the scores of these two principal components did not show an obvious and consistent trend. This may have been caused by interference from water or the systematic environment. This might have also occurred because the scores of each component used for mapping only contained information about the original independent variables, without taking into account the relationship between independent and dependent variables [26], which reduced the model’s robustness and prediction capability [27,28,29]. A second PCA of the spectra, within the range 1200–1500 cm^−1^, was conducted after deconvolution. The results showed that PC1 of the high-concentration group reached 99.52% and that of the low-concentration group was 99.39% (Figure 3a,b, respectively). The NO_3_^−^–N concentration showed a regular distribution in the PC1 area, wherein the plot shifted to positive values of PC1 as the NO_3_^−^–N concentration of the solution increased.

### 3.3. Prediction of Nitrate Nitrogen in Water with Water Deduction

**Figure 4:**
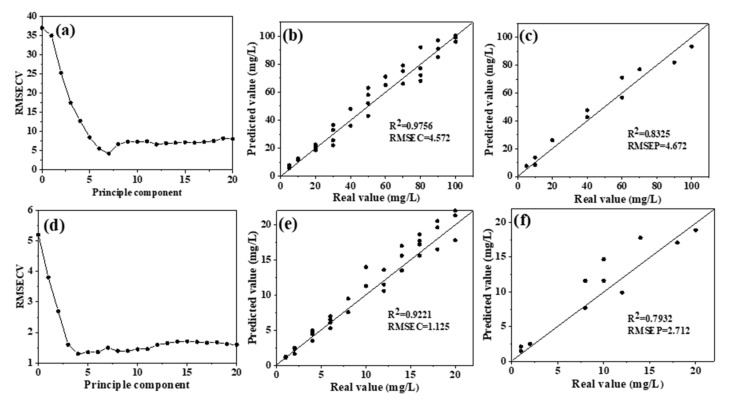
Distribution and model evaluation of the partial least squares (PLS) factor (**a**,**d**), training set ((**b**,**e**); *n* = 33), and testing set ((**c**,**f**); *n* = 11) of the partial least squares regression (PLSR) prediction model (without water deduction) for high nitrate solutions (**a**–**c**) and low nitrate solutions (**d**–**f**). Note: *RMSECV*, root mean square error in cross validation; *RMSEC*, root mean square error of calibration; *RMSEP*, root mean square error of prediction.

PLSR was used to model the 1200–1500 cm^−1^ region of the spectra, and the overall dataset was divided into a training set (75% of the overall set) and a testing set (25% of the overall set) using random division. The cross-validation method was used to determine the optimal number of PLS factors. As shown in Figure 4a, the optimal number of PLS factors in the high-concentration group was 7, which corresponds to the minimum of *RMSECV* [13]; therefore, the first seven PLS factors were used to construct the PLSR model. Figure 4b,c show the distributions of the real and predicted values of the training and testing sets, respectively. The linear regression coefficient (*R*^2^) of the measured and predicted values of NO_3_^−^–N in the training set was 0.9756, representing a significant correlation. The *R*^2^ of the prediction set was 0.8325, and the *RPD* value was 1.86 (Table 1).

*RPD* is an important model evaluation parameter in infrared spectrum analysis; it is the ratio of the standard deviation (*SD*) of a sample to the root mean square error (*RMSE*). Generally, when *RPD* > 1.8, quantitative detection can be conducted. An *RPD* between 2 and 2.5 indicates a good quantitative prediction model, while one higher than 3 suggests excellent model prediction performance [30,31]. Therefore, this model can be applied for the rapid quantitative determination of high NO_3_^−^–N concentrations in water bodies. The optimal number of PLS factors in the low-concentration group was 4 (Figure 4d); thus, the PLSR model was built using the first four factors. The distributions of the true and predicted values of the training and testing sets are shown in Figure 4e,f, respectively. The evaluation index (*R*^2^) of the training set was 0.9221, suggesting a significant correlation. However, the *R*^2^ of the testing set was much lower at only 0.7932 and *RPD* also decreased to 1.75, which is lower than the minimum standard of 1.8 for quantitative detection. These results show that the predictive performance of this model is poor and that the model is not suitable for detecting low concentrations of NO_3_^−^–N in water.

### 3.4. Prediction of Nitrate Nitrogen in Water with Deconvolution (without Water Deduction)

**Figure 5:**
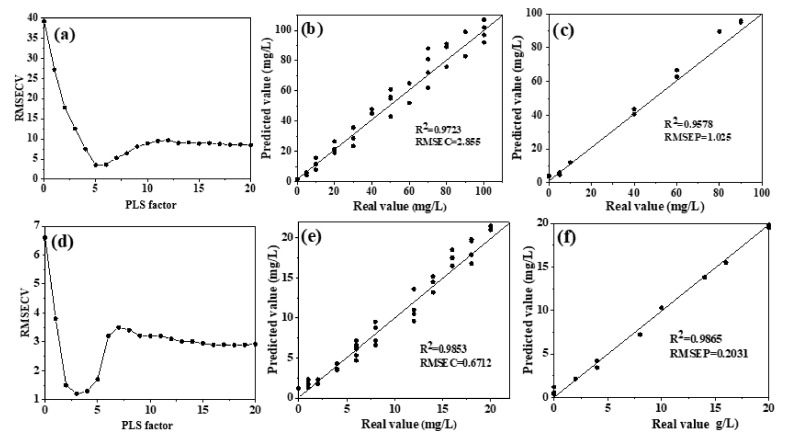
Distribution and model evaluation of the PLS factor (**a**,**d**), training set ((**b**,**e**); *n* = 36), and testing set ((**c**,**f**); *n* = 12) of the PLSR prediction model with deconvolution (without water deduction) for high nitrate solutions (**a**–**c**) and low nitrate solutions (**d**–**f**). Note: *RMSECV*, root mean square error in cross validation; *RMSEC*, root mean square error of calibration; *RMSEP*, root mean square error of prediction.

Similarly, PLSR was also used to model the characteristic bands of NO_3_^−^–N, within the 1200–1500 cm^−1^ region, obtained by deconvolution curve-fitting. The overall dataset was divided into a training set (75% of the overall set) and a testing set (25% of the overall set) using random division. Cross-validation was used to obtain the optimal number of principal components in the high- and low-concentration groups and then, to establish PLSR models. For the high-concentration group, the optimal number of PLS factors was 5 (Figure 5a). The *R^2^* of real and predicted values of nitrate nitrogen in the training set and testing set were 0.9723 and 0.9578 (Figure 5b,c), respectively, implying a significant correlation. The *RPD* value was 4.22 (Table 1), which was higher than 3, suggesting that the model had an excellent predictive capability. The optimal number of principal components in the low-concentration group model was 3 (Figure 5d), the correlation coefficients (*R^2^*) in the training set and testing set were 0.9853 and 0.9865 (Figure 5e,f), respectively, and the *RPD* was 3.15 (Table 1), indicating an excellent predictive performance. The above results showed that in both concentration groups, the PLSR model established based on spectra deconvolution (without water deduction) achieved better performance than the model established with water deduction, which indicated that deconvolution peak-fitting could effectively reduce water interference to extract useful spectral information.

In the process of linear multivariate calibration analysis, the limit of detection (LOD) could be estimated by 3*σ* or 3*σ*/*m* [32]. σ was the standard deviation of the predicted concentration, which could be replaced with *RMSE*, and m was the fitting-curve slope of the model (using the real value as the *X*-axis and the predicted value as the *Y*-axis). The *m* value of the high concentration group model was 0.6236, and the *RMSEP* was 1.025 (Figure 5c), so the 3*σ* was 3.075 and the 3*σ*/*m* was 4.931. In the low concentration group, the *m* was 0.7122, and *RMSEP* was 0.2031 (Figure 4f); therefore, the 3*σ* and 3*σ*/*m* were 0.6039 and 0.8491, respectively.

## 4. Conclusions

In this study, combined with the PLSR model, FTIR-ATR spectroscopy was applied to detect nitrate in high- and low-concentration solutions, with deconvolution algorithm (without water deduction) comparing with conventional water deduction algorithms. In both the high- and low-concentration groups, the PLSR model based on the non-deduction of water (deconvolution curve-fitting) performed significantly higher prediction accuracy than the model established by deducting water to quantitatively predict nitrate nitrogen, which provided a more effective analysis method for the rapid determination of different concentrations of nitrate in water bodies.

## Figures and Tables

**Table 1:** Number of optimal PLS factors and model evaluation results of the PLSR model with and without water deduction.

Treatment	Statistical Parameters	High-Concentration Group	Low-Concentration Group
Water deduction	Correlation coefficient (*R*^2^)	0.8325	0.7932
	*RPD*	1.86	1.75
	Optimized PLS factor	7	4
Without water deduction	Correlation coefficient (*R*^2^)	0.9578	0.9865
	*RPD*	4.22	3.15
	Optimized PLS factor	5	3

## Supplementary Materials

The following are available online. Figure S1: The process of deconvolution, Figure S2: Second derivative of nitrate absorption. (a), high-concentration group with the range of 0–100 mg L^−1^; (b), low-concentration group with the range of 0–20 mg L^−1^. Figure S3: The equilibrium of electron clouds in nitrate.
