# Supplementary material for: In Situ Determination of Nitrate in Water Using Fourier Transform Mid-Infrared Attenuated Total Reflectance Spectroscopy Coupled with Deconvolution Algorithm
Source: Molecules. 2020 Dec 10;25(24):5838. doi: 10.3390/molecules25245838 (PMC7764078; doi:10.3390/molecules25245838)
Supplement: Supplementary file 1 [file molecules-25-05838-s001.pdf]

Supplementary

In Situ Determination of Nitrate in Water Using Fourier Transform Mid-Infrared Attenuated Total Reflectance Spectroscopy Coupled with Deconvolution Algorithm

Fangqun Gan[1,2], Ke Wu[2], Fei Ma[1] and Changwen Du[1,3,*]

1 Institute of Soil Science Chinese Academy of Sciences, Nanjing 210008 China; fma@issas.ac.cn
2 College of Environment and Ecology, Jiangsu Open University, Nanjing, 210017 China; qunfanggan@163.com
3 University of Chinese Academy of Sciences, Beijing 100049, China; chwdu@issas.ac.cn
* Correspondence: chwdu@issas.ac.cn; Tel: +86-25-86881565

Deconvolution of FTIR-ATR spectra of nitrate

The spectra in Figures 2c and 2d were extracted from the raw spectra (Figures 1a and 1b) with the algorithm of deconvolution, and the data treat process and the algorithm parameters were showed in Figure S1.

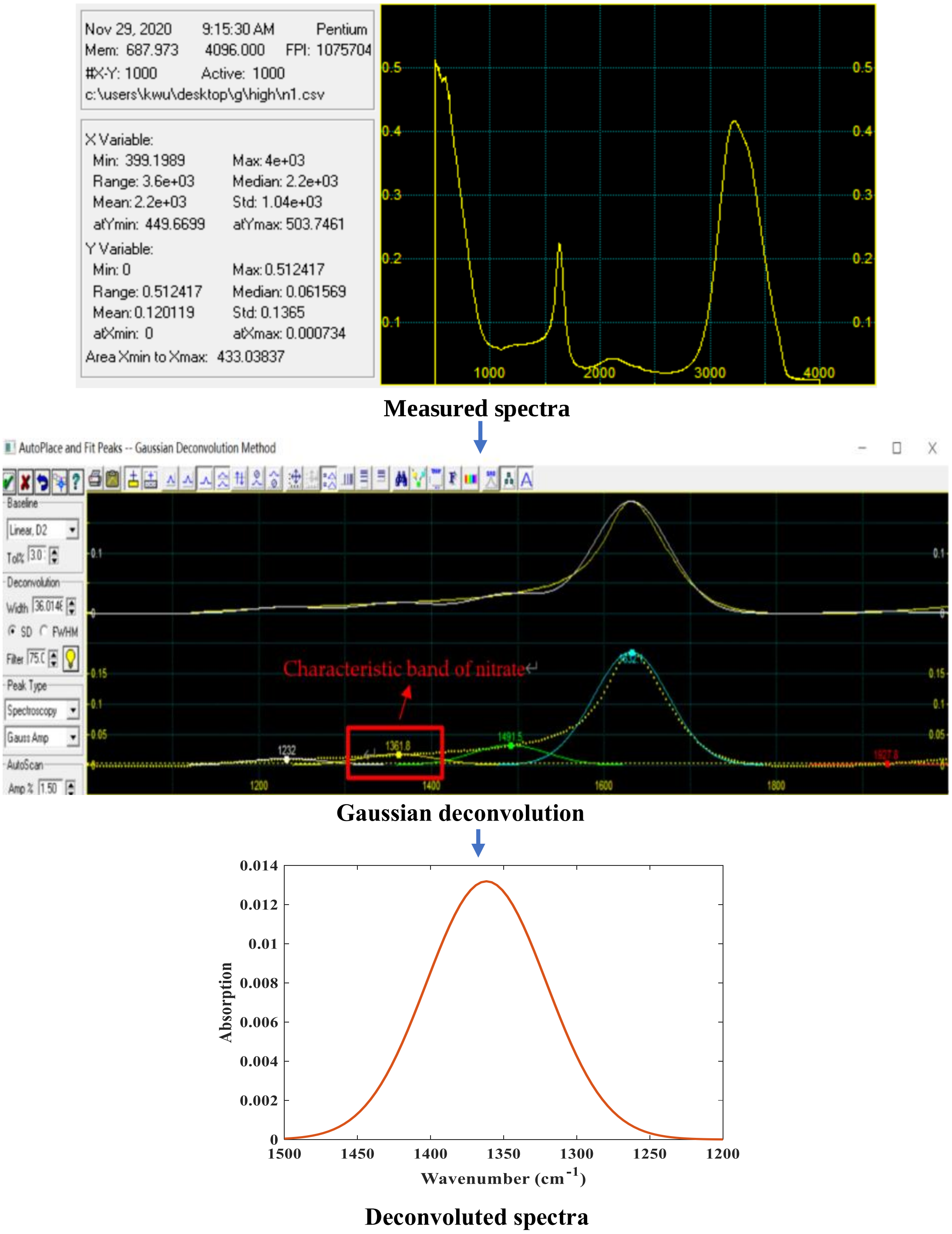

Figure S1. The process of deconvolution

In order to minimize the signal interference from water (wavenumber range of 1500-2000 cm^{-1}), the spectrum in the range of 1000-2000 cm^{-1} were deconvoluted. The nitrate absorption band was more susceptible to systematic environment as well as water interference due to a small target signal. Therefore, the parameters of baseline, bands, deconvolution width, filter and peak type (etc.) were optimized and set before the deconvolution process, so that the characteristic absorption bands of nitrate with fixed wavenumbers were obtained.

The second derivative of nitrate absorption was also calculated (Figure S2). Several bands at various wavenumber (1200-1500 cm^{-1}) were associated with N-O vibration resulted from the re-distribution of electron clouds in nitrate, respectively (Figure S3). In deconvolution process, the sum of squares due to error (SSE) of spectral data varied from 0.005938 to 0.007065, indicating a good deconvolution effect.

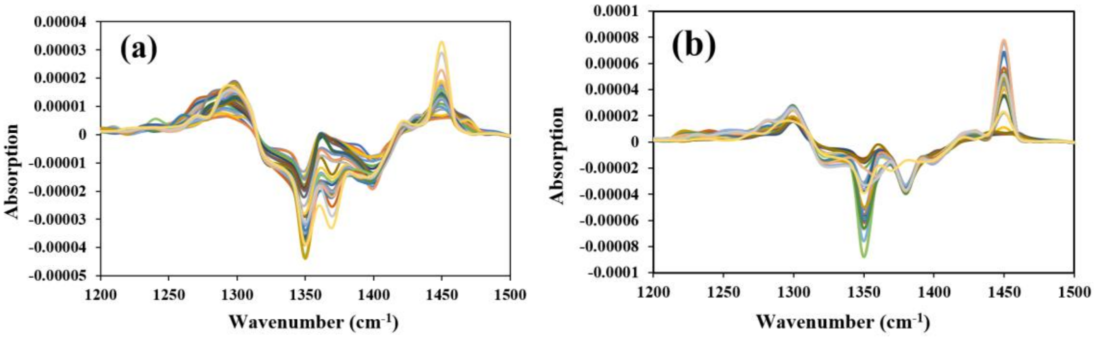

Figure S2. Second derivative of nitrate absorption. a, high-concentration group with the range of 0-100 mg L^{-1}; b, low-concentration group with the range of 0-20 mg L^{-1}.

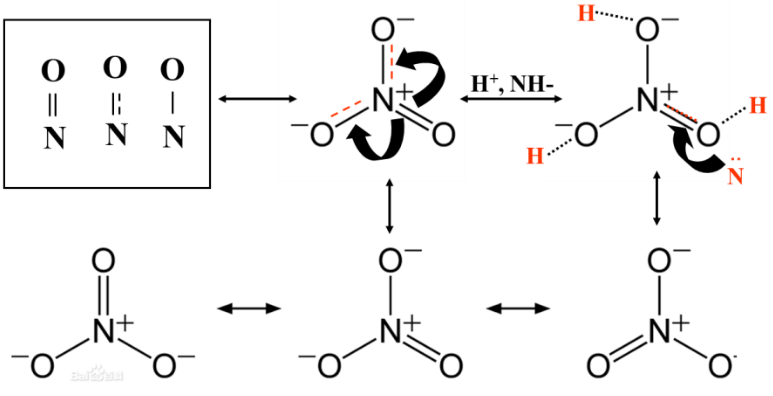

Figure S3. The equilibrium of electron clouds in nitrate
